# Scale up and pharmacokinetic study of a novel mutated chimeric tissue plasminogen activator (mt-PA) in rats

**DOI:** 10.1038/srep43028

**Published:** 2017-02-22

**Authors:** Mozhgan Raigani, Mohammad-Reza Rouini, Ali-Akbar Golabchifar, Esmat Mirabzadeh, Behrouz Vaziri, Farzaneh Barkhordari, Fatemeh Davami, Fereidoun Mahboudi

**Affiliations:** 1 Biotechnology Research Center, Pasteur Institute of Iran, Pasteur Avenue, Tehran, Iran; 2 Biopharmaceutics Division, Department of Pharmaceutics, Faculty of Pharmacy, Tehran University of Medical Sciences, Tehran 14155/6451 Iran; 3 Department of human vaccines and sera, Razi Vaccine and Serum Research Institute, Agricultural Research, Education and Extension Organization, Karaj, Iran

## Abstract

Because of high mortality caused by cardiovascular diseases, various fibrinolytic agents with diverse pharmacokinetic and pharmacodynamic properties have been developed. A novel mutated chimeric tissue plasminogen activator (mt-PA) was developed by the removal of first three domains of t-PA, insertion of GHRP sequence and mutation towards resistance to plasminogen activator inhibitor-1 (PAI-1). Mt-PA protein was expressed in Expi293F cells. The expression level of mt-PA was found to be 5000 IU/mL. Following purification, the pharmacokinetic properties of mt-PA were evaluated in three doses in rats. Data related to mt-PA were best fitted to two compartment model. With the increase in dose, the Area Under the plasma concentration-time Curve (AUC_0→∞_) increased. The elimination half-life (t_1/2_) of mt-PA was in the range of 19.1–26.1 min in three doses while that of Alteplase was 8.3 min. The plasma clearance (CLp) of mt-PA ranged from 3.8 to 5.9 mL/min in three doses, which was several times lower than that of Alteplase (142.6 mL/min). The mean residence time (MRT) of mt-PA ranged from 23.3–31.8 min in three doses, which was 4–5 times greater than that of Alteplase (6 min). Mt-PA showed extended half-life and mean residence time and is a good candidate for further clinical studies.

Cardiovascular diseases, caused by disorders of heart and blood vessels, account for 17.3 million deaths per year that is expected to grow to more than 23.6 million by 2030^1^,^2^. In 2011, the estimated annual costs of cardiovascular diseases and stroke amounted to a total of more than $320.1 billion^1^. Thrombolytic drugs particularly plasminogen activators (PAs) play an essential role in this respect and PAs can clear circulatory occlusions due to fibrin clot or thrombus. PAs convert plasminogen to the active serine protease plasmin which, in turn, dissolves fibrin, the insoluble matrix of clots^3^.

Tissue-type plasminogen activator (t-PA) is one of the fibrin-specific serine proteases that plays a crucial part in the fibrinolytic system^4^,^5^. T-PA is composed of a single chain polypeptide of 527 amino acids and includes 17 disulfide bridges^6^. The mature form of t-PA comprises five distinct domains: a finger domain (F) involved in the high-affinity binding of t-PA to fibrin and hepatic clearance of t-PA^7^, an epidermal growth factor-like domain (EGF) which contributes to the hepatic clearance of t-PA^8^, a kringle 1 domain (K1) which is important in the uptake of t-PA by mannose receptors on liver cells^9^, a K2 domain involved in the high-affinity binding to fibrin and activation of plasminogen, and a serine protease domain (S) where the catalytic activity of t-PA takes place^10^.

The main inhibitor of t-PA is PAI-1, a member of the serpin family (serine-protease inhibitor), which plays its role as a pseudo-substrate for target serine proteases^11^. PAI-1 is synthesized by endothelial cells and hepatocytes, and partially by the α-granules of platelets^12^. Similarly, plasmin is inhibited mainly by α_2_-antiplasmin, yet plasmin-bounded fibrin is never inhibited^6^.

Because of the short plasma half-life (4–6 min) of Alteplase^13^, a large dose is required to obtain therapeutic blood levels, which in turn may lead to higher bleeding and re-occlusion risks due to a decreased plasma fibrinogen level^14^. Therefore, through deleting or substituting the sequence of Alteplase genes, mutants of PAs with diverse pharmacokinetic and pharmacodynamic properties have been developed to treat thrombotic diseases^15^,^16^.

Reteplase (rPA) is a non-glycosylated deletion mutant of t-PA with prolonged half-life (14–18 min), in which the F, EGF, and K1 domains of wild-type t-PA have been deleted. Since the F domain was deleted, the affinity of Reteplase to fibrin was significantly (5 fold) lower than that of Alteplase. Accordingly, Reteplase causes more fibrinogen depletion than Alteplase^17^.

The decrease in the plasma clearance, in Tenecteplase (TNK-mutant of Alteplase), was made by site directed mutagenesis at positions 117 and 103 in the K1 domain (half-life of 17–20 min). In addition, 4 amino acids KHRR (296–299 position) bound to PAI-1 were substituted with alanine, which made resistance of Tenecteplase to PAI-1^18^.

In our mt-PA, the three (F, EGF, and K1) domains of t-PA were removed in order to decrease the plasma clearance and increase the half-life of recombinant protein in the circulating blood. Moreover, a chimeric tetra-peptide Gly-His-Arg-Pro (GHRP) which has high affinity for fibrin was added to the upstream of K2S in order to make up for F domain deletion effect^19^. Furthermore, to prevent PAI-1 inhibition, the four amino acids bound to PAI-1 at positions 296–299 were replaced by four alanine amino acids^20^. Therefore, we expected a novel mt-PA with better properties compared to other plasminogen activators.

Transient gene expression (TGE) is used for the expression of monoclonal antibodies and recombinant proteins in which suspension adapted mammalian cells, especially human embryo kidney-293 (HEK-293) and Chinese Hamster Ovary (CHO) cell lines, are commonly applied^21^. Because of the advances in the rapid production of g/L quantities of recombinant proteins without time investment, TGE is a widely used method in pre-clinical and clinical studies^22^.

In the current study, the scale up of mt-PA, previously developed in lab scale^19^, was performed by TGE system and its pharmacokinetic properties were investigated in rats and compared to wild-type t-PA (Alteplase).

## Results

### Transfection into Expi293F cells and the determination of transfection efficiency

**Figure 1:**
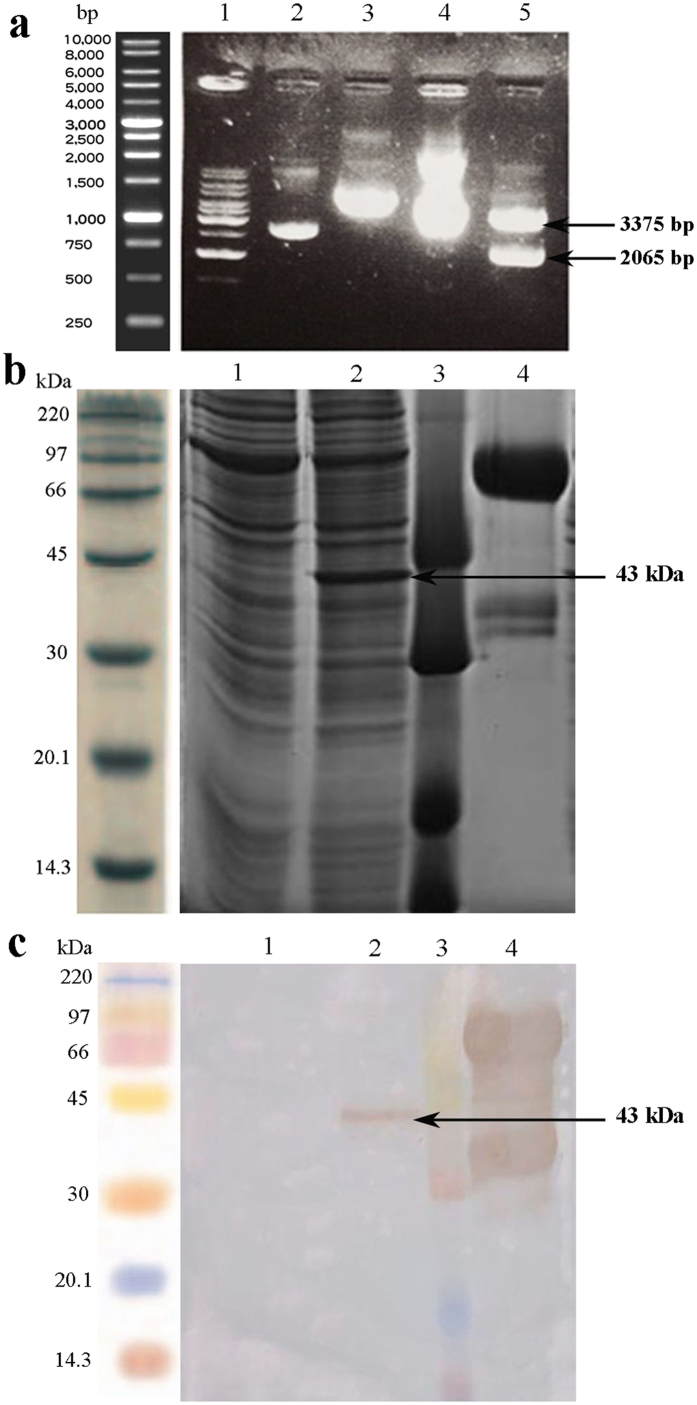
Gel-electrophoresis, SDS-PAGE and Western blotting analysis of mt-PA. (**a**) Gel-electrophoresis analysis of the mt-PA- pTracer-SV40 Plasmid by *Sma*I restriction enzyme digestion on 1% agarose gel. (Lane 1) DNA Ladder 1 Kb. (Lane 2) pTracer-SV40 Plasmid without the mt-PA gene. (Lane 3) pTracer-SV40 Plasmid without the mt-PA gene digested by *Sma*I restriction enzyme. (Lane 4) pTracer-SV40 Plasmid with the mt-PA gene. (Lane 5) pTracer-SV40 Plasmid with the mt-PA gene digested by *Sma*I restriction enzyme. (**b**) The mt-PA expression analysis in transfected Expi293F cells supernatant on a 12% SDS-PAGE gel. (Lane 1) Supernatant from non-transfected Expi293F cell culture medium as negative control. (Lane 2) The mt-PA Expi293F cell culture medium. (Lane 3) Rainbow Molecular Weight Marker (code RPN756). (Lane 4) Alteplase as positive control. (**c**) Western blotting detection of the mt-PA expression in the supernatant of transfected Expi293F cells. (Lane 1) Supernatant from non-transfected Expi293F cell culture medium (negative control). (Lane 2) The mt-PA Expi293F cell culture medium. (Lane 3) Rainbow Molecular Weight Marker (code RPN756). (Lane 4) Alteplase (full length form). The samples in **a** (gel) and **b** (blot) derived from the same experiment and the gel and blot were processed in parallel.

The presence of mt-PA was confirmed by *Sma*I restriction enzyme in the mt-PA pTracer-SV40 plasmid, and further corroborated in Fig. 1a.

The mt-PA pTracer-SV40 plasmid was transfected into Expi293F cells by ExpiFectamine™ 293 transfected reagent. In flow cytometry, non-transfected cells were first gated and set as the system threshold and 24, 48 and 72 h following transfection, the transfection rates measured 42%, 61% and 86% on the green fluorescent protein (GFP) basis, respectively (data not shown). The transfection efficiency was also confirmed by fluorescent microscope.

### Analysis of expression via sodium dodecyl sulfate-polyacrylamide gel electrophoresis (SDS-PAGE) and Western blotting procedure

After the detection of the protein expression on days 3, 5, 6, 7 and 9, the maximum protein production belonged to the fifth day of incubation. Supernatants harvested from transiently transfected Expi293F cells were analyzed by SDS-PAGE on 12% polyacrylamide gel and Western blotting. As shown in Fig. 1b, the 43 kDa protein bond belongs to mt-PA indicated in lane 2, and no bond is seen in the negative control (lane 1). Lane 4 demonstrates the full-length t-PA protein, which has 65 kDa. The expression of mt-PA was ultimately confirmed by Western blotting analysis (Fig. 1c).

### Quantitative analysis

The mt-PA activity in the supernatant samples of transfected Expi293F cells was quantitatively determined by an ELISA-based biofunctional immunosorbent assay, Biopool Chromolize t-PA Assay Kit. The activity (IU/mL) of the tissue plasminogen activator was calculated from the standard curve. The expression of mt-PA was 5000 IU/mL.

### Purification

**Figure 2:**
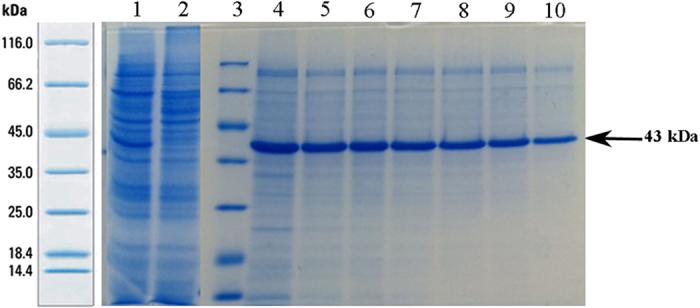
The analysis of the affinity chromatography fractions of the transfected Expi293F cells supernatant on SDS-PAGE gel. (Lane 1) Prior to the purification of supernatant. (Lane 2) Flow- through. (Lane 3) Unstained Protein MW Marker (Thermo Scientific). (Lane 4–10) The fractions of second elution buffer. The fractions of first elution buffer was cropped. The full-length gel is presented in Supplementary Figure S1.

After the production of pilot scale and filtration of culture supernatant through a 0.45 μm membrane filter, affinity purification was conducted by the foregoing buffers (Fig. 2). As shown in Fig. 2, lanes 4–10 are related to the fractions of second elution buffer. The fractions of first elution buffer can also be found as Supplementary Figure S1. Mt-PA was purified through the first step of purification.

### Confirmation of mt-PA after the second step of purification

**Figure 3:**
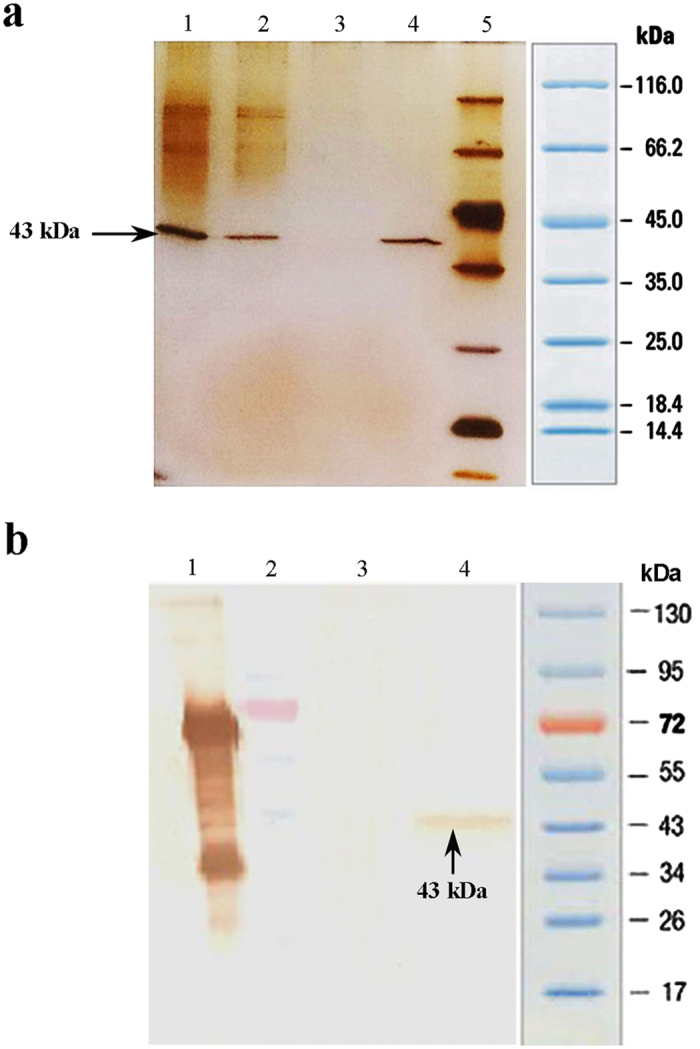
SDS-PAGE and Western blotting analysis of mt-PA after purification. (**a**) Detection of mt-PA after the 2nd step of purification using SDS-PAGE procedure. (Lane 1) Before size exclusion chromatography. (Lane 2) First peak. (Lane 4) Mt-PA. (Lane 5) Unstained Protein MW Marker (Thermo Scientific). (**b**) Western blotting analysis of formulated purified mt-PA. (Lane 1) Alteplase as positive control. (Lane 2) Prestained Protein Marker (Fermentas). (Lane 3) Supernatant from non-transfected Expi293F cell culture medium (negative control). (Lane 4) Formulated purified mt-PA with 43 kDa size.

In the second step of the purification, i.e. size exclusion chromatography, all proteins were separated based on their molecular weight. The fractions of each peak were analyzed by SDS-PAGE procedure according to Fig. 3a. As shown, lane 4 belongs to mt-PA, found after gel filtration chromatography. After size exclusion chromatography, a highly purified recombinant protein was achieved.

### Western blotting analysis of formulated pure protein

Purified mt-PA was later subjected to formulation by ultrafiltration technique. After concentrating the protein, SDS-PAGE and Western blotting detection were performed according to Fig. 3b. Finally, the purity of the formulated protein was confirmed with 43 kDa bond. Also, based on quantitative analysis via Chromolize t-PA Assay Kit, formulated mt-PA was found to be 5000 IU/mL.

### Pharmacokinetic analysis of mt-PA and Alteplase

**Figure 4:**
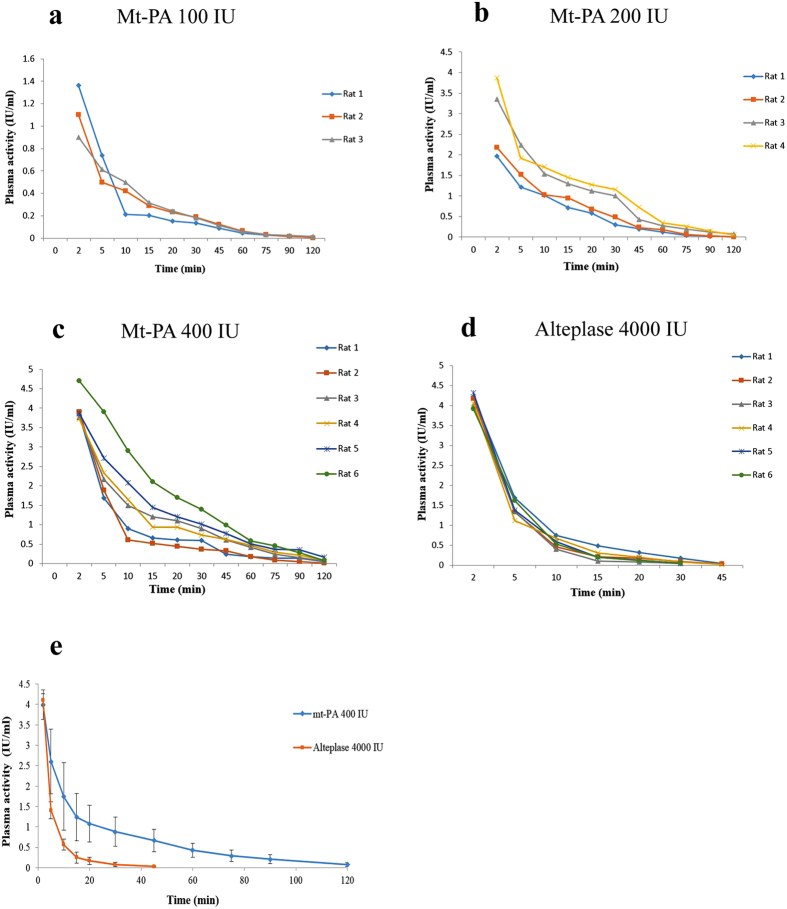
Plasma activity of mt-PA and Alteplase after their intravenous administration to rats. (**a**) Plasma activity of a 100 IU mt-PA after i.v. bolus injection to three rats. (**b**) Plasma activity of a 200 IU mt-PA after i.v. bolus injection to four rats. (**c**) Plasma activity of a 400 IU mt-PA after i.v. bolus injection to six rats. (**d**) Plasma activity of a 4000 IU Alteplase after i.v. bolus injection to six rats. (**e**) The comparison of mean (Mean ± SD) plasma activity of 400 IU mt-PA and 4000 IU Alteplase after intravenous administration to rats (n = 6).

Figure 4 shows the plasma activity-time curve after the intravenous (i.v.) administration of 100, 200 and 400 IU mt-PA and 4000 IU Alteplase to rats. Furthermore, Fig. 4e compares the plasma activity of 400 IU mt-PA and 4000 IU Alteplase after the intravenous administration to six rats in each group.

The plasma activity of both compounds decreased in a biphasic manner. The pharmacokinetic parameters were calculated by a two-exponential model (Table 1). With the increase in dose, AUC_0→∞_ increased in rats. The half-life (t_1/2_) of mt-PA in β phase was longer than that of Alteplase. CLp of mt-PA was much lower than that of Alteplase. In the three implemented doses of mt-PA, the volumes of distribution at steady state (V_ss_) were lower than that of Alteplase. The distribution volume of the central (V_1_) and the peripheral (V_2_) compartment in mt-PA were also lower than that of Alteplase. Furthermore, the area under the first moment curve (AUMC) of mt-PA was several times greater than that of Alteplase. The elimination rate constant (K_10_) of mt-PA was almost 0.09 ± 0.03 min^−1^ in comparison to Alteplase where the same constant was found to be 0.3 ± 0.03 min^−1^. Finally, MRT of mt-PA was 4–5 times greater than that of Alteplase. Based on these figures, it can be concluded that after administration, mt-PA has a longer presence in plasma than Alteplase.

Table 2 shows calculated pharmacokinetic parameters of different tissue-type plasminogen activators in rats and rabbits. The estimated parameter, their standard errors and relative standard errors and *p*-values obtained from the Wald test (only for the coefficients of the covariates) shown in Table 3.

**Figure 5:**
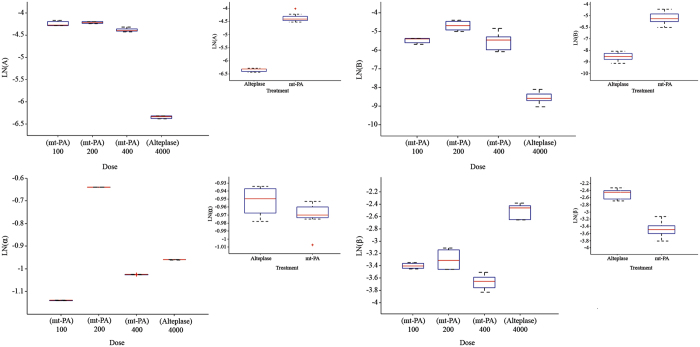
Box plot of parameter model estimates of individual values over different treatment and dosing amount were determined from a pharmacokinetics model in rat. *Abbreviations:* A, the y-intercept of the distribution phase; B, the y-intercept of the elimination phase; α, the distribution slope; β, the elimination slope.

Box plot of parameter model estimates of individual values over different treatment and dosing amount were determined from a pharmacokinetics model in rat (Fig. 5).

## Discussion

Thrombolytic drugs such as plasminogen activators play essential parts in the treatment of cardiovascular diseases or strokes caused by circulatory occlusions^1^. In this regard, developing new t-PA variants with superior pharmacokinetic and pharmacodynamics properties compared to full-length t-PA is of great interest. As mentioned, in this study, the F, E, and K1 domains of t-PA were deleted in mt-PA such as Reteplase. The principal focus of these efforts was to reduce the size of molecule and increase the circulatory half-life of mt-PA by eliminating certain regions. To enhance fibrin affinity which is reduced by the deletion of the above mentioned domains^3^, additional fibrin-binding sites such as GHRP sequence was inserted into the molecule^19^. Higher fibrin specificity could increase potency, and decrease bleeding^23^. Furthermore, deletion variants of t-PA (Reteplase) are not resistant to PAI-1inhibition^16^. The mutation in amino acids (296–299) in Tenecetepalse from KHRR to AAAA, could resist PAI-1 inhibition^24^.

As it is illustrated, presence of the mt-PA gene into the pTracer-SV40 plasmid was confirmed by *Sma*I restriction enzyme (Fig. 1a) resulting in one fragment when the mt-PA gene is absent, and two fragments when it is included. The protein migrated with an apparent molecular mass of 43 kDa on SDS-PAGE; the same bond was observed with Western blotting detection (Fig. 1c). According to the results in Fig. 3b, the presence of the purified mt-PA protein was confirmed after 2 step purification and formulation processes by Western blotting technique.

Mt-PA was expressed in Expi293F cells in order to acquire a high-level production of recombinant protein using TGE procedure. As mentioned, various kinds of expression systems exist, yet mammalian cells are still the essential hosts for the industrial production of therapeutic recombinant proteins. Because of the rapid rather than stable production of recombinant proteins, TGE is generally employed for the transient production of high quantities of recombinant proteins^22^. The expression level was found to be 5000 IU/mL, where the expression is promising compared to the enzymatic activity of t-PA in E. coli (3–7 IU/mL)^25^, nonmodified rCHO (50 IU/mL)^3^, or Leishmania tarentolae (70 IU/mL)^26^.

As shown in Fig. 3a, highly purified mt-PA was obtained after the 2 step purification procedure (lane 4). Affinity purification was carried out through the repeated alteration of pH and NaCl concentration. Following size exclusion chromatography, HPLC, as an analytical method, was performed so as to confirm the purity of mt-PA, where the purity percentage of protein was proven acceptable. To increase the accuracy of the acquired bond, we supplanted Coomassie brilliant blue staining method with silver nitrate staining.

In this study, the pharmacokinetic properties of mt-PA were investigated in rats and were compared with wild-type t-PA. The characterization of mt-PA pharmacokinetics mainly focused on the measurement of plasma activity levels. Three doses of mt-PA, including; 100, 200 and 400 IU were intravenously administered to rats in the experimental groups. 4000 IU of Alteplase and the formulation buffer were also intravenously administered to rats as positive and negative control, respectively.

The data obtained in rats in the present study showed a good fit to two compartment models and presented very promising pharmacokinetic characteristics. The mt-PA distributed to peripheral compartment with the same distribution half-life (almost 1.8 min, *p* > 0.05) as Alteplase. However, the elimination half-life of mt-PA was approximately 22 min in three doses which is quite longer than Alteplase (8.3 min, *p* < 0.05) (Table 1). A half-life of approximately 4.3–12.7 min was reported after the i.v. administration of a novel recombinant plasminogen activator BM 06.022 developed by Martin and colleagues^27^; the half-life related to the administration of alteplase, on the other hand, varied from 1.0 to 2.6 min (Table 2). BM 06.022 contains only the kringle 2 and protease domains of wild-type t-PA^27^. The calculated CLp of mt-PA did not show dose-dependency and amounted to 3.8 to 5.9 mL/min which in comparison to Alteplase (142.6 ± 15.2 mL/min) showed a much slower clearance from plasma. The findings of the present study demonstrate that our recombinant tissue-type plasminogen activator mt-PA has a three-fold longer half-life and several times slower clearance rate than that of Alteplase in rats after i.v. bolus injection. A previous study showed that the systemic clearance of BM 06.022 was lower than t-PA (7.6–5 mL/min/kg versus 43–17 mL/min/kg) in rats^27^.

As shown in Table 1, the results confirm that after bolus administration, AUC of mt-PA increased with the increase in dose and was higher than that of Alteplase. V_ss_ in mt-PA was considered to be several times lower than that of Alteplase, inspite of the lower intravenous administration dose in mt-PA. Furthermore, the elimination rate constant (k_10_) of mt-PA decreased in comparison to Alteplase. Finally, MRT of mt-PA increased 4-5 times more than that of Alteplase. In conclusion, the results indicate that after intravenous administration, mt-PA, compared with Alteplase, has a longer presence in plasma.

A longer half-life accompanied by smaller volume of distribution would allow single-bolus administration to keep a higher plasma concentration of mt-PA, which would result in a longer fibrinolytic activity and a less frequent drug administration.

A similar outcome was reported by Martin and colleagues^28^ who developed a deletion variant of BM 06.021 consisting only of the kringle 2 and protease domains of human t-PA. They compared a pharmacokinetic study of the new variant with Alteplase in the rabbit upon i.v. bolus injection. They proved that the half-life of BM 06.021 was about 2.7 times longer compared with Alteplase and its clearance was lower than that of Alteplase (Table 2). They illustrated that the longer elimination half-life and lower clearance of BM 06.021 was related to the elimination of the first three domains^28^.

Two different receptors are known to be related to the uptake of t-PA in the liver: the mannose receptor located on liver endothelial cells and the low density lipoprotein-related protein (LRP/α_2_-MRreceptor) located on liver parenchymal cells, which are most important for the hepatic clearance of t-PA^9^. Furthermore, Camani showed that LRP essentially recognizes finger and/or EGF domains of t-PA^29^.

Several researchers have found that the high-mannose-type oligosaccharide at Asn117 in K1 domain is recognized by the mannose receptors on liver endothelial cells^30^,^31^. They explained that the reduced clearance and increased half-life of t-PA protein may be due to the decline of uptake through the mannose receptor in the liver by the removal of the high mannose glycosylation in K1 domain^32^. The longer half-life and lower plasma clearance in our study could also be accounted for via the deletion of K1 domain which decreases the uptake of mt-PA by the mannose receptor.

In another study, one point mutation was created in wild-type t-PA, resulting in Gln117 t-PA^33^. They demonstrated that CLp of Gln117 t-PA in rats was almost 2.6-fold lower than that of WT t-PA. Moreover, MRT of Gln117 t-PA was approximately 4.8-fold greater than that of WT t-PA (Table 2). In this case, the absence of the high mannose oligosaccharide at Asn117 could only decrease the hepatic clearance of t-PA and did not affect the extrahepatic clearance of t-PA^33^.

Pamiteplase and rt-PA parameters in rats were calculated by a two compartment model. However, the three-exponential model analysis well fitted the plasma concentration of both compounds and was comparable to parameters calculated by non-compartmental model; accordingly, two compartment model was considered as suitable for rat pharmacokinetics^34^. Our results also show that all observed activities of mt-PA in all three studied doses suitably fit the two compartment model and calculated AUCs are comparable to non-compartmental model.

In this study, treatment effects were tested using Wald test. Level of significance (*p*-value) for covariates coefficients as shown in Table 3 demonstrated significant difference (β, A, B in two compartment pharmacokinetic model) between two treatments (Alteplase and mt-PA). No significant difference was found in pharmacokinetic parameters in three doses of mt-PA. So, treatment found to significantly affect pharmacokinetic parameters (β, A, B).

We continued to examine the rats for up to one month after the injection of compounds and collect blood samples in order to investigate the toxicity effects on rats. The results guaranteed that the compounds did not have side effects and no mortality was observed in rats.

The risk of bleeding and re-occlusion considerably increase in Reteplase due to the elimination of finger domain. To compensate, GHRP sequence, previously analyzed in lab scale study, was added to our mutated t-PA; the results suggested that fibrin affinity of mt-PA was superior to that of full-length t-PA^19^. However, this field is open to further researches in the future. In addition, PAI-1 inhibition of Reteplase is similar to t-PA, indicating that the activity of Reteplase can be prevented by PAI-1. In contrast to Reteplase, mt-PA is more resistant to PAI-1 than t-PA. The higher resistance to PAI-1 could be translated to potential for increased therapeutic potency compared to full length form, particularly with respect to the lysis of platelet-rich clots^23^. Also, reducing PAI-1-mediated re-occlusion after thrombolysis is a process yet to be investigated.

In conclusion, mt-PA has modified pharmacokinetic parameters compared to Alteplase, meaning mt-PA exhibits a long biological half-life and a low clearance rate and resistance to PAI-1 as well as a high affinity to fibrin. Therefore, it can be a suitable plasminogen activator with reduced molecular size and better pharmacokinetic properties, which can anticipate therapeutic dosage arrangement with an intravenous injection in humans.

## Methods

### All methods were performed in accordance with the relevant guidelines and regulations

#### Rats

Female wistar rats, aged 12–14 weeks, weighing 230–250 gr, were obtained from laboratory animals department of Pasteur Institute of Iran-Karaj. The animals were (1) acclimatized for one week in a group condition prior to the experiments, (2) fed with a standard laboratory pellet and clean water, provided adlibitum, and (3) maintained under standard condition of temperature (22 ± 2), humidity (50–55%) and light (12 h light/12 h dark cycles). This experimental study was approved by the Ethics Committee of Pasteur Institute of Iran. The ethics code (IR.PII.REC.1395.42) was allocated for our project, because of approving the experiments. All procedures were performed in accordance with the ethical Helsinki standards. The rats were randomly divided into three groups, namely positive control, negative control and experimental.

### Preparation of the expression plasmid

The expression vector containing the cDNA of mt-PA (mt-PA pTracer-SV40) was gifted from Dr. Davami^19^. The mt-PA pTracer-SV40 plasmid was first confirmed by *Sma*I restriction enzyme (Fermentas, Lithuania) digestion and then purified using EndoFree Plasmid Giga kit (Qiagen Germany).

### Cell culture

The 293 cell line is a stable line established by primary embryonal human kidney transformed with sheared human adenovirus type 5 DNA. The E1A adenovirus gene expressed in 293 cells participates in the transactivation of certain viral promoters, allowing these cells to produce very high levels of protein. The Expi293F™ cells (GIBCO, Life Technologies, USA) have been adapted to serum-free, suspension culture in Expi293™ Expression Medium from Invitrogen (GIBCO Invitrogen, USA). The media were formulated with GlutaMAX™-I reagent and incubated at 37 °C in 5–8% CO_2_.

### Transfection into Expi293F cells and determination of transfection efficiency

In a cell density of 2.5 × 10^6^ cells/mL with a final volume of 1 mL, the mt-PA pTracer-SV40 plasmid was transfected to the cells by ExpiFectamine™ 293 reagent, the cationic lipid-based transfection reagent, with a 1:2.7 DNA to reagent ratio following the manufacturer’s recommendation (Expi293™ Expression System Kit). The negative control group was also comprised of one plate of non-transfected cells. Transfection efficiency was calculated either by flow cytometry (CyFlow, Partec, Germany) or manually, using a fluorescent microscope (BEL, Italy). In all experiments, the emitted fluorescence intensity was analyzed after 24, 48 and 72 h of transfection with excitation and emission at 488 and 509 nm wavelengths, respectively. In flow cytometry, viable cells were gated and quantitatively analyzed by FloMax software for GFP expression. In the manual method, the ten views of each sample were visually counted. Finally, the average fluorescent-counts in each experiment were compared with non-transfected cells.

### SDS-PAGE and Western blotting analysis

Supernatants were harvested from transient transfected Expi293F culture and analyzed by electrophoresis on a 12% polyacrylamide gel using Coomassie brilliant blue staining method according to the Laemmli^35^. Western blotting was also analyzed based on Sambrook *et al*.^36^ using a semidry blotting system (Bio-Rad, USA). Polyclonal rabbit anti-t-PA antibody (1/1000 dilution, Abcam, USA) and HRP conjugated goat anti-rabbit antibody (1/2500 dilution, Santa Cruz. USA) were used as primary and secondary antibodies, respectively. Ultimately, protein bands were visualized by adding DAB solution (Sigma-Aldrich, Germany).

### Amidolytic activity test

The mt-PA activity in the culture medium was quantitatively determined by amidolytic activity test using Chromolize t-PA Assay Kit^24^ (Trinity Biotech plc, Ireland). The test is based on bio-functional immunosorbent assay during which t-PA is captured by strip-coated sp-322 monoclonal antibody. The kit was utilized based on manufacturer’s protocol, which is briefly explained. T-PA standards and the samples were added to the microtest strip wells. After discarding the test plasma and standards, the wells were washed with a mild detergent. 50 μl of plasminogen and substrate reagent were then added to each well, resulted in yellow color, read at 405 and 492 nm wavelengths. The amount of color developed was proportional to the amount of t-PA activity in the sample. Absorbance of each sample at 492 nm was subtracted from 405 nm and the activity (IU/mL) was calculated through standard values (0, 0.5, 1, 1.5 and 2 IU/mL).

### Scale up and protein expression in pilot scale

The Expi293F cells were transiently transfected in the pilot scale. Supernatants obtained from transfected Expi293F culture were centrifuged at 4 °C and purified by a subsequent filtration through a 0.45 μm membrane filter.

### Purification

#### Affinity purification

The purification procedure was performed using HiTrap Benzamidine FF (high sub) column^37^. The column is specified for the purification of serine proteases. The binding and wash buffers were as follow: 0.5 M NaCl, 0.05 mM Tris-HCL, pH 7.4. Two elution buffers were performed using a step gradient of 1 M NaCl, 10 mM HCl (pH 2.0) and 0.05 M Glycine with a pH of 3.0 where mt-PA was eluted from the column primarily with 1 M NaCl, 10 mM HCl (pH 2.0) elution buffer and then, with 0.05 M Glycine. The purification steps were performed based on the manual. With the pH and conductivity of these buffers, the t-PA electrostatically bound to the column while other non-serine protease proteins did not bind to the resin and were removed in the column flow through.

### Size exclusion chromatography purification

Size exclusion chromatography (AKTApurifier, GE Healthcare Life Sciences, USA) was performed so as to achieve a highly pure protein^38^. The chromatographic procedure was carried out using XK26-100 column packed with superdex 200 which had been equilibrated with phosphate buffer, pH 7.0. The employed flow rate was 2.2 mL/min. The results were analyzed by UNICORN software. And the fractions of gel filtration were investigated for the desired protein using SDS-PAGE procedure.

### High performance liquid chromatography (HPLC) as an analytical method

The purity of the recombinant protein was analyzed by HPLC (KNAUER, Germany) size exclusion chromatography. The column was packed with silica-based rigid, hydrophilic gel in internal diameter. The protein sample was loaded onto the column and the mobile phase, with a flow rate of 0.5 mL/min, containing 30 g/L of sodium dihydrogen phosphate and 1 g/L of sodium dodecyl sulfate, adjusted to pH 6.8 with diluted sodium hydroxide solution. The procedure was performed based on BP protocols. The results were detected by a spectrophotometer set at 214 nm and the acquired data were analyzed by ChromGate software.

### Formulation

Formulation buffer was prepared, firstly containing 34.8 g/L of arginine, and 0.1 g/L of polysorbate 80 (pH 7.4). The 10 kDa MW cutoff filter was used for ultrafiltration (Merck Millipore, Germany) and concentration. Finally, the purity of mt-PA was analyzed by 12% SDS-PAGE, followed by Coomassie blue staining and Western blotting analysis. Moreover, the activity of the purified protein was analyzed by amidolytic activity test.

### Pharmacokinetic analysis

#### Preparation and administration of dosing solution

A lyophilized formulated vial containing 50 mg of Alteplase was dissolved in water for injection to make an Alteplase stock solution (1 mg/mL). The dilution was performed to achieve 4000 IU per 0.5 mL for i.v. bolus injection. The activity of the formulated mt-PA solution was also detected and the solution itself was watered down in order to obtain 100, 200 and 400 IU in 0.5 mL for i.v. bolus injection. So as to eliminate the confounding effects of formulation reagents, the formulation buffer (carrier) was injected to three rats as negative control.

In the three mt-PA groups, mt-PA was administered at 100 IU (three rats), 200 IU (four rats) and 400 IU (six rats); Alteplase was injected to one group (six rats) at 4000 IU.

In the mt-PA groups, blood samples of 360 μL were taken prior to, and 2, 5, 10, 15, 20, 30, 45, 60, 75, 90 and 120 min after the injection of mt-PA. In Alteplase group, blood samples (360 μL) were obtained before, and 2, 5, 10, 15, 20, 30 and 45 min following Alteplase injection. And in the negative control group, blood samples (360 μL) were achieved before, and 2, 5 and 45 min after buffer administration^34^.

### Blood sampling

The animals were anesthetized by intraperitoneal administration of ketamine (100 mg/kg) + Xylazine (5 mg/kg). The tip of the tail was severed and the first sample of blood was collected. After the intravenous administration of plasminogen activators into the tail vein of rats^13^, blood samples were transferred periodically from the tail vein in Eppendorf tubes containing 40 μL of 3.8% sodium citrate, mixed gently, and centrifuged at 1800 × g for 10–15 min at 4 °C. Plasma was immediately removed from pellet and stored frozen at −70 °C until assayed^33^.

### Specifying the activity of plasminogen activators

The activity of plasminogen activators (mt-PA and Alteplase) in plasma samples was measured by amidolytic activity test.

### Calculation of pharmacokinetic parameters

After a single intravenous administration, the observed plasma t-PA activity curves were fit to a two compartment open model using the nonlinear least-squares regression program as follows:


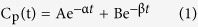


where C_p_(t) is the concentration of mt-PA or Alteplase in the plasma at time t. A and B are the y-intercepts of the distribution and elimination phases, which have slopes of α and β respectively. D is the administered dose and k_10_ is the first-order elimination rate constant from the central compartment. k_12_ and k_21_ are the rate constants for drug transfer between the central and the peripheral compartments. The distribution volume of the central compartment (Vc = V_1_), V_ss_, CLp, MRT and AUMC were calculated by the following equations^33^:


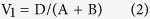



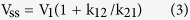



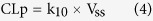



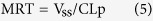



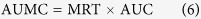


Furthermore, categorical covariates treatment (Alteplase and mt-PA) and dose of mt-PA were added using equation


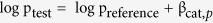


where P represent median covariate value for pharmacokinetic parameter and β is the coefficient of the covariate effect.

### Statistical analysis

The data, reported as mean ± SD, were statistically analyzed using SPSS 16; A *P* < 0.05 was taken to be statistically significant.

## Additional Information

**How to cite this article**: Raigani, M. *et al*. Scale up and pharmacokinetic study of a novel mutated chimeric tissue plasminogen activator (mt-PA) in rats. *Sci. Rep.* **7**, 43028; doi: 10.1038/srep43028 (2017).

**Publisher's note:** Springer Nature remains neutral with regard to jurisdictional claims in published maps and institutional affiliations.

## Supplementary Material

Supplementary Figure S1

## Figures and Tables

**Table 1:** Pharmacokinetic parameters of Alteplase and mt-PA after the intravenous administration of 100, 200 and 400 IU mt-PA and 4000 IU Alteplase to rats.

Parameters	Alteplase (n = 6)	Mt-PA (n = 13)		
	4000 IU	100 IU	200 IU	400 IU
t_1/2_ (α) (min)	1.8 ± 0.03	1.85 ± 0.05	1.82 ± 0.02	1.83 ± 0.02
t_1/2_ (β) (min)	8.3 ± 1.3	20.3 ± 1.4	19.1 ± 3.8	26.1 ± 3.4
K_10_ (min^−1^)	0.3 ± 0.03	0.11 ± 0.03	0.08 ± 0.02	0.09 ± 0.03
K_12_ (min^−1^)	0.07 ± 0.02	0.18 ± 0.01	0.16 ± 0.01	0.19 ± 0.02
K_21_ (min^−1^)	0.12 ± 0.01	0.12 ± 0.03	0.18 ± 0.01	0.12 ± 0.03
AUC _0→∞_ (IU.min/mL)	28.3 ± 3.3	17 ± 1.6	59.2 ± 23.9	83.3 ± 30.8
AUMC (min.min.IU/mL)	173.6 ± 63.9	398.2 ± 78.2	1608.0 ± 940.3	2749.4 ± 1301.0
V_ss_ (mL)	838.0 ± 117.6	137.6 ± 5.2	91.2 ± 17.7	163.9 ± 39.9
V_1_ (mL)	509.3 ± 13.7	54.6 ± 7.2	48.3 ± 8.4	58.7 ± 4.5
V_2_ (mL)	328.7 ± 111.8	82.9 ± 9.2	42.9 ± 9.3	105.2 ± 36.7
CLp (mL/min)	142.6 ± 15.2	5.9 ± 0.6	3.9 ± 1.6	5.4 ± 2.1
MRT (min)	6 ± 1.5	23.3 ± 2.5	25.4 ± 5.7	31.8 ± 6.1

*Abbreviations*: t_1/2_ (α), the distribution half-life; t_1/2_ (β), the elimination half-life; K_10_, the first-order elimination rate constant; k_12_ and k_21_, the elimination rate constants between the central and the peripheral compartments; AUC_0→∞_, the Area Under the plasma concentration-time Curve; AUMC, the area under the first moment curve; V_ss_, the volumes of distribution at steady state; V_1_, the distribution volume of the central compartment; V_2_, the distribution volumes of the peripheral compartment; CLp, the plasma clearance; MRT, the mean residence time. Each parameter was presented as Mean ± SD; n = 3–6/dose.

**Table 2:** Calculated pharmacokinetic parameters of different tissue-type plasminogen activators.

t-PA	Dose	t_1/2_	Number of compartment	AUC	V_ss_	Vc	CL_total_	MRT	Ref.
	**(mg/kg)**	**α β γ (h)**		**(ng. h/mL)**	**(l/kg)**		**(l/kg/h)**	**(h)**	
Pamiteplase^*^	0.03	0.15, 0.55, 3.59	Three	174.5	0.30		0.17	1.76	34
	0.1	0.22, 0.82, 6.30	Three	550.7	0.23		0.18	1.29	
	0. 3	0.06, 0.56, 9.46	Three	1427.8	0.37		0.21	1.75	
Alteplase^*^	0. 3	0.02, 0.09, 1.16	Three	211.3	0.18		1.42	0.13	
	**(mg/kg) activity**	**λ1, λ2 (h)**		**(μg.h/mL)**	**(l/kg)**	**(l/kg)**	**(mL/min/kg)**		
DSPA^*^	3	0.1, 0.9	Two	4.9	0.3	0.1	10.3		39
	10	0.1, 0.8	Two	23.0	0.14	0.06	7.3		
	30	0.1, 1.4	Two	76.7	0.10	0.08	6.8		
		α β (min)		(IU.h/mL)			(mL/min/kg)		
BM 06.021^**^	200 kU/kg	5.6 ± 2.6, 17.6 ± 1.5	Two	452 ± 47			7.5 ± 0.8		28
Alteplase^**^	200 kU/kg	2.1 ± 0.3, 10.9 ± 1.0	Two	133 ± 18			22.2 ± 3.1		
		**α β (min)**		**(IU.h/mL)**			**(mL/min/kg)**		
BM 06.022^*^	200 kU/kg	4.3 ± 0.9, 12.3 ± 1.7	Two	456 ± 40			7.6 ± 0.6		27
Alteplase^*^	200 kU/kg	1.0 ± 0.1, 14.7 ± 3.0	Two	92 ± 14			43 ± 7.8		
				**i.v., i.p.v.(% of dose·min/mL)**	**(mL/kg)**	**(mL/kg)**	**(mL/min/kg)**	**(min)**	
Gln117 t-PA^*^	250 μg/kg		Two	18.73 ± 3.27, 17.27 ± 1.97	393.4 ± 90.7	105.2 ± 17.9	17.1 ± 4.1	22.9 ± 0.1	33
WT t-PA^*^	250 μg/kg		Two	9.52 ± 1.10, 5.21 ± 1.40	212.9 ± 41.1	112.8 ± 17.6	44.5 ± 11.1	4.8 ± 0.3	

*Abbreviations*: T-PA, tissue-type plasminogen activator; t_1/2_, half-life; AUC, the Area Under the plasma concentration-time Curve; V_ss_, the volumes of distribution at steady state; V_C_, the distribution volume of the central compartment; CL_total_, total clearance; MRT, the mean residence time; DSPA, Desmodus rotundus plasminogen activator; i.v., Femoral vein; i.p.v., Portal vein. ^*^Rats, ^**^Rabbits.

**Table 3:** Parameter estimates of two compartment pharmacokinetic model for Alteplase and mt-PA.

Parameter		s.e.	r.s.e.(%)	β_cov (Alteplase,mt-PA dose)_		*p*-value^*^	β_cov (Alteplase,mt-PA)_		*p*-value^*^
α (Dose = 4000*)	0.383	0.03	8						
α (Dose = 100)	0.32	0.064	20	β_(α,Dose=100)_	−0.18	0.4			
α (Dose = 200)	0.527	0.19	36	β_(α,Dose=200)_	0.32	0.39			
α (Dose = 400)	0.358	0.044	12	β_(α,Dose=400)_	−0.0658	0.65	β_(α,Dose=mt-PA)_	−0.0362	0.79
β (Dose = 4000*)	0.0811	0.0074	9						
β (Dose = 100)	0.0331	0.0029	9	β_(β,Dose=100)_	−0.897	<1E-010			
β (Dose = 200)	0.0367	0.0025	7	β_(β,Dose=200)_	−0.794	<1E-010			
β (Dose = 400)	0.0254	0.0015	6	β_(β,Dose=400)_	−1.16	<1E-010	β_(β,Dose=mt-PA)_	−0.981	<1E-010
A (Dose = 4000*)	0.00176	0.00015	9						
A (Dose = 100)	0.0144	0.0028	19	β_(A,Dose=100)_	2.1	<1E-010			
A (Dose = 200)	0.0148	0.0061	41	β_(A,Dose=200)_	2.13	4.4E−07			
A (Dose = 400)	0.0124	0.0016	13	β_(A,Dose=400)_	1.95	<1E-010	β_(A,Dose=mt-PA)_	1.96	<1E-010
B (Dose = 4000*)	0.000191	4.E-05	21						
B (Dose = 100)	0.00415	0.00089	21	β_(B,Dose=100)_	3.08	<1E-010			
B (Dose = 200)	0.00922	0.0016	17	β_(B,Dose=200)_	3.88	<1E-010			
B (Dose = 400)	0.00402	0.00058	14	β_(B,Dose=400)_	3.05	<1E-010	β_(B,Dose=mt-PA)_	3.31	<1E-010

*Abbreviations*: α, the distribution slope; β, the elimination slope; A, the y-intercept of the distribution phase; B, the y-intercept of the elimination phase. *P-values obtained from the Wald test only for the coefficients of the covariates.
