# Supplementary material for: Scale up and pharmacokinetic study of a novel mutated chimeric tissue plasminogen activator (mt-PA) in rats
Source: Sci Rep. 2017 Feb 22;7:43028. doi: 10.1038/srep43028 (PMC5320447; doi:10.1038/srep43028)
Supplement: Supplementary Figure S1 [file srep43028-s1.pdf]

Scale up and pharmacokinetic study of a novel mutated chimeric tissue plasminogen activator (mt-PA) in rats

Mozhgan Raigani[1], Mohammad-Reza Rouini[2], Ali-Akbar Golabchifar[3], Esmat Mirabzadeh[1], Behrouz Vaziri[1], Farzaneh Barkhordari[1], Fatemeh Davami[1] & Fereidoun Mahboudi[1]

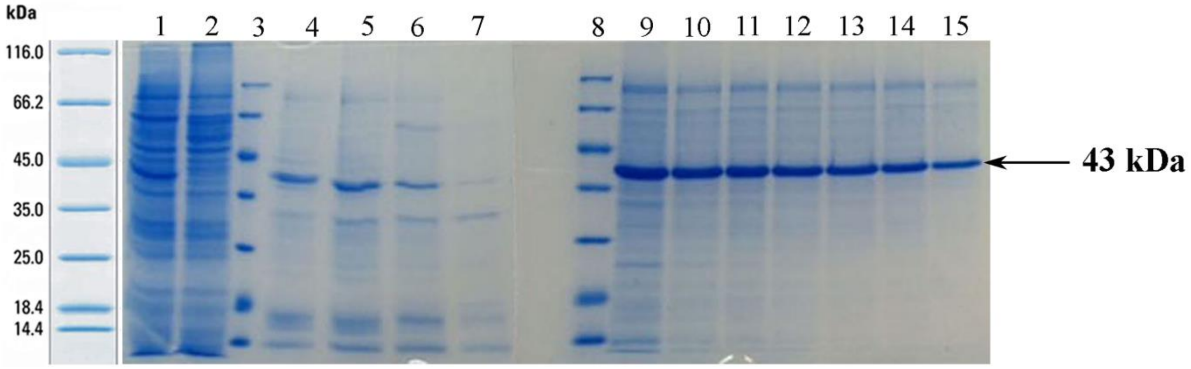

Supplementary Figure S1 The analysis of the affinity chromatography fractions of the transfected Expi293F cells supernatant on SDS-PAGE gel. (Lane 1) Prior to the purification of supernatant. (Lane 2) Flow- through. (Lane 3, 8) Unstained Protein MW Marker (Thermo Scientific). (Lane 4-7) The fractions of first elution buffer. (Lane 9-15) The fractions of second elution buffer.
